# The Presence of Chondral Pathology Is Not Associated With an Increase in Meniscal Root Retear, Reoperation, or Subsequent Total Knee Arthroplasty Rates

**DOI:** 10.1002/ars2.70043

**Published:** 2026-05-27

**Authors:** Matthew D. Benson, Richard J. VanTienderen, Austin C. Benson, Benjamin D. Packard, Steven M. Leary, Kyle R. Duchman, Brian R. Wolf, Robert W. Westermann

**Affiliations:** ^1^ The University of Iowa Carver College of Medicine Iowa City Iowa U.S.A.; ^2^ University of Iowa Health Care Iowa City Iowa U.S.A.

## Abstract

**Purpose:**

To identify whether chondral pathology is associated with an increase in failure rates of isolated meniscal root repairs.

**Methods:**

We retrospectively reviewed meniscus repairs from 2012 to 2022 at the author's institution. Nonroot repairs and cases with total or same compartment meniscectomy, cartilage restoration, osteotomy, or other concurrent ligament repair or reconstruction were excluded. Chondral pathology was defined as any abnormal finding in the articular cartilage of the medial, lateral, or patellofemoral compartments. We defined repair failure as clinical evidence of retear with magnetic resonance imaging confirmation, subsequent reoperation, or conversion to total knee arthroplasty (TKA). No minimum follow‐up time was established, but each case was followed to the most recent pertinent orthopaedic follow‐up. Fisher's two‐tailed exact test was used to determine significance.

**Results:**

Sixty‐three isolated meniscus repairs (63 medial, 0 lateral) were included, of which 51 (81.0%) had existing chondral pathology. Five patients (7.9%) underwent reoperation, six (9.5%) had evidence of retear, and three (4.8%) had a subsequent TKA. Medial tears accounted for 100% (6/6) of retears. There was a failure rate of 11.8% (6/51) for repairs with existing chondral pathology. There was no significant difference in clinical retear rate (11.8% vs 0%, *P* = 1.000), reoperation rate (9.8% vs 0%, *P* = 1.000), or subsequent TKA rate (5.9% vs 0%, *P* = .476) with or without existing chondral pathology at the time of repair, respectively. 100% (3/3) of subsequent TKAs had existing chondral pathology at the time of repair.

**Conclusions:**

In this study, we did not find a statistically significant difference in meniscal root retear, reoperation, or subsequent TKA rates with or without existing chondral pathology.

**Level of Evidence:**

Level IV, retrospective therapeutic case series.

## Graphical abstract

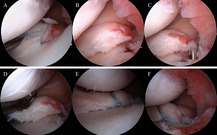

The meniscus is a critical component of the fully functioning knee joint. Functionally, the meniscus provides both chondral protection and contributes to knee stability.^1^ The meniscus is composed of fibrocartilage, which contains proteoglycans, glycoproteins, and high water content, and this structure enables the meniscus to withstand axial loading, rotational forces, and resist compression.^2^ The meniscus is also known to provide nutrition to the surrounding cartilage and reduce friction.^2^ When the meniscus is damaged, it can compromise normal knee function.

Meniscus injuries can result in significant disability, limiting overall knee function.^3^ Meniscal root tears are a particular type of meniscal tear that can be defined as an avulsion injury or radial tear occurring within 1 cm of the bony tibial attachment, whereas nonroot tears occur further away from the tibial attachment sites.^3^ A normally functioning meniscus is able to convert and disperse axial tibiofemoral loads into radial tangential stress, known as hoop stress.^4^ A meniscal root tear results in a loss of these hoop stresses, which leads to an increased load on the articular cartilage, decreased tibiofemoral contact area, and increased peak pressures. Biomechanically, this is equivalent to a total meniscectomy and has been found to rapidly accelerate chondral degeneration.

When conservative management is contraindicated or proves ineffective,^1^ surgery, in the form of partial to total meniscectomy versus repair, is indicated.^5^ Successful meniscus repair improves knee function while also restoring the chondral protection and stabilizing role of the native meniscus.^6^ In the setting of a meniscal root tear, operative intervention is often recommended early due to the rapid progression of chondral injury associated with nonoperative management.

Tears of the posterior root of the medial meniscus are commonly associated with concomitant chondral pathology, often degenerative in nature.^7^ There is limited evidence regarding the effect cartilage defects have on the success rate of meniscal root repair surgery. The purpose of this study was to identify whether chondral pathology is associated with an increase in failure rates of isolated meniscal root repairs. The hypothesis was that existing cartilage defects will be associated with increased isolated meniscus root repair retear rates.

## METHODS

A retrospective chart review of isolated meniscal procedures was performed between January 1, 2012, and December 31, 2022, at the author's institution, after obtaining institutional review board approval [IRB: 202304511 (Isolated Meniscus Repair)]. The Current Procedural Terminology code 29882 was used to identify subjects. Exclusions were made for cases involving nonroot repairs, total or same compartment meniscectomy, cartilage restoration (osteochondral autograft transplantation, osteochondral allograft, particulated cartilage implantation), osteotomy, or other concurrent ligament repair or reconstruction. Key variables recorded included age, sex, body mass index (BMI), laterality (right vs left), location of tear (medial vs lateral), surgeon, root repair technique, associated procedures, presence of cartilage defects which were subclassified by compartment (medial, lateral, or patellofemoral), and rehabilitation protocol (Table 1).

**TABLE 1:** The Author's Institution's Meniscal Root Repair—Physical Therapy Rehabilitation Guidelines

Post‐op Visit Timeframe	Weight Bearing	Brace	ROM	Therapeutic Exercises
Initial post‐op—2 wk	TTWB	Locked in extension for ambulation, removed for ROM and exercises	0° to 90°	Straight leg raises, ankle range of motion
2 to 6 wk	TTWB	Locked in extension for ambulation, removed for ROM and exercises	0° to 90°	PROM to tolerance, patella mobilization, quadriceps/hamstring/glute sets, hamstring stretches, hip strengthening, SLR
6 to 12 wk	FWB	None	Progress to full and pain free	PROM‐AROM
				Patella mobilization
				Quadriceps/hamstring strengthening
				Stationary bike for ROM when flexion reached 110°
				Continue to advance lower extremity strengthening activities
				Avoid twisting while weight bearing and avoid squat beyond 90°
				Progress ROM to full
				Add proprioception training
				Closed kinetic chain exercises
12 to 16 wk	FWB	None	Full	Advance above activities
				Progress to jogging and gradual return to sports specific training and exercises if all symptoms have resolved and full ROM obtained

*Note*: Post‐op Visit Timeframe Determines Degree of Weight Bearing (TTWB, FWB, or PWB), Joint Range of Motion (ROM—AROM and PROM), and Special Exercises, Like SLR. AROM, active ROM; FWB, full weight bearing; Post‐op, Post‐operative; PROM, passive ROM; PWB, partial weight bearing; ROM, joint range of motion; SLR, single leg raise; TTWB, toe‐touch weight bearing; wk, week.

**FIGURE 1:**
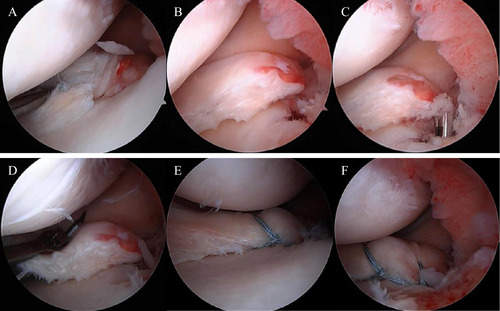
(A) Arthroscopic view of a medial meniscus posterior horn white‐white longitudinal split extending to a complete radial tear within 1 cm of the posterior root, with instability showed with a probe. (B) Arthroscopic view of a medial meniscus posterior horn after debridement with meniscal biters with a prepared root site tunnel on the right. (C) Arthroscopic view of a medial meniscus root tear and a transtibial tunnel with an arthroscopic instrument emerging intra‐articularly from an anteromedial incision portal. (D) Arthroscopic view of the medial meniscus posterior horn with intra‐articular suture passing device showing good meniscal tissue capture. (E) Arthroscopic view of a reduced posterior meniscal horn in the previously debrided footprint. (F) Arthroscopic view of a posterior meniscal horn fixated by two luggage‐tag sutures shuttled down the transosseous tunnel.

Indications for surgery involved joint line/posterior knee pain and/or mechanical symptoms corresponding with the presence of a root tear identified on magnetic resonance imaging (MRI) evaluation. The surgical root repair technique was consistent among all cases and completed with a trans‐osseous technique with 2 to 3 luggage tag sutures (Figure 1). A standardized meniscal root repair physical therapy rehabilitation protocol was consistent among all cases: with nonweight bearing or toe‐touch weight bearing for the first 6 weeks in a knee brace locked in extension for ambulation and allowing range of motion (ROM) from 0° to 90° when seated, followed by progression to weight bearing and ROM as tolerated after 6 weeks (Table 1).

Cartilage defects were defined as any abnormal finding in the condylar cartilage of the compartment before the repair (i.e., erosion, full‐thickness lesions, damage, superficial fraying, superficial loss, or chondromalacia). Degenerative vs trauma etiology was not recorded. We defined repair failure as any clinical evidence of retear with MRI confirmation or subsequent ipsilateral knee reoperation (reoperation rate), including subsequent conversion to total knee arthroplasty (TKA) on the ipsilateral knee (Table 2). No minimum follow‐up time was established, but each case was followed to the most recent pertinent orthopaedic follow‐up.

**TABLE 2:** Isolated Meniscus Root Repairs With and Without Existing Cartilage Defects (Medial, Lateral, or Patellofemoral) at the Time of Repair (n = 63) That Had Clinical Evidence of a Retear With MRI Confirmation, Subsequent Reoperation, or Subsequent TKA

	Cartilage Defect		No Cartilage Defect	
Retear (yes/no)∗	6	45	0	12
Reoperation (yes/no)∗∗	5	46	0	12
TKA (yes/no)∗∗∗	3	48	0	12

MRI, magnetic resonance imaging; TKA, total knee arthroplasty.

* *P* = 1.000.

** *P* = 1.000.

*** *P* = .476.

### Statistical Analysis

All data was recorded using REDCap software. SPSS statistical software was used to complete Fisher's two‐tailed exact tests when comparing meniscal root retear, reoperation, and subsequent TKA rates with or without existing chondral pathology, with significance set to *P* < .05. An unpaired Welch's t‐test was used to compare the average BMI between repairs with and without cartilage defects and repairs that failed with those that have not, with a significance set to *P* < .05.

## RESULTS

**FIGURE 2:**
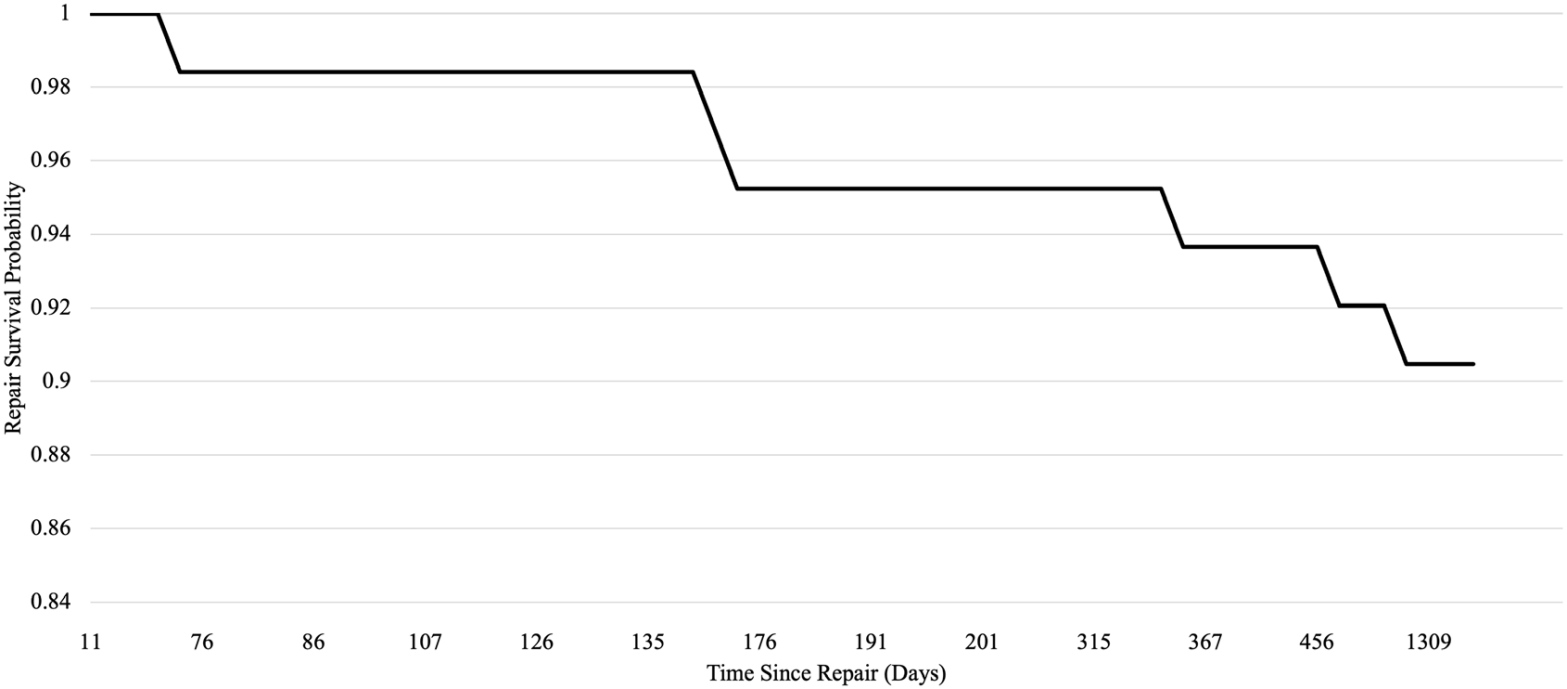
Kaplan‐Meier survival curve showing the probability of isolated meniscus root repair survival over time (n = 63). The y‐axis represents cumulative repair survival probability, and the x‐axis represents time to the most recent pertinent orthopaedic follow‐up visit in days (range = 11‐1987 days).

Five hundred seventy‐four isolated meniscal procedures were performed during the 10‐year period at our institution. After applying inclusion and exclusion criteria, sixty‐three isolated meniscus root repairs (63 medial, 0 lateral) were included, of which 74.6% (47/63) had an associated procedure completed concurrently (Table 3). Mean age was 48.08 ± 11.94 years; 71.4% (45/63) were female with a mean BMI of 34.56 ± 6.63, and 39.7% (25/63) involved the right leg. The average number of days to the last follow‐up visit was 279.3 (range 11‐1987). There was a total of four sports medicine fellowship‐trained orthopaedic surgeons, each completing between 9 and 29 procedures. Of the 63 patients, 6 (9.5%) had evidence of retear, 5 (7.9%) underwent reoperation, and 3/63 (4.8%) had a subsequent TKA. Cumulative failure rate was 9.5% (6/63; Figure 2). Two of 6 (33.3%) failed repairs were female, and 100% of those females showed evidence of retear and underwent reoperation, but 1/2 (50%) of them had a subsequent TKA. Four of 6 (66.7%) of failed repairs were male, and 100% of those males showed evidence of retear, but 3/4 (75%) underwent reoperation, and 2/4 (50%) had a subsequent TKA. The average number of days to failure was 354.7 (range 58‐911), with 108.5 (range 58‐159) for females and 477.8 (range 171‐911) for males. The average number of days to TKA was 384.3 (range 215‐558), with 380 (range 380) for females and 386.5 (range 215‐558) for males. Medial tears accounted for 100% (6/6) of retears, lateral for 0% (0/6). The average BMI of failed repairs (36.69 ± 7.29) was not significantly different than the average BMI of successful repairs to date (34.34 ± 6.59, *P* = .478). The average BMI of repairs with cartilage defects present at the time of repair (34.88 ± 6.94) was not significantly different than the average BMI of the repairs without cartilage defects at the time of repair (33.20 ± 5.10, *P* = .351).

**TABLE 3:** The Number and Type of Nonexcluded Associated Procedures (n = 47) That Were Completed Concurrently With the Root Repairs of This Study (n = 63)

	Total	Total Cases With at Least 1 Associated Procedure	Synovectomy	Chondroplasty	Partial Lateral Meniscectomy	Microfracture	Cyst Excision	Medial Release	Lateral Release	Reverse Notchplasty
Successful repair procedures	57	41	18	12	12	7	1	1	1	1
Failed repair procedures	6	6	1	0	3	4	1	0	0	0

Fifty‐one of the 63 (81.0%) repairs had existing chondral pathology at the time of surgery. Forty‐nine of those 51 patients (96.1%) had chondral pathology involving the medial weight bearing area of the knee condyle (Table 4). Thirty‐eight of the 45 (84.4%) female and 13 of the 18 (72.2%) male repairs had existing chondral pathology at the time of surgery. Of those with chondral pathology, 36 of the 38 (94.7%) females and 13 of the 13 (100%) males had the medial weight bearing area of the knee (condyle) affected. A full description describing the distribution of chondral pathology is provided in Table 4. Twelve of the 63 (19.0%) did not have any chondral pathology noted, which was 7 of the 45 females (15.6%) and 5 of the 18 (27.8%) males. Repairs with existing chondral pathology had a clinical retear rate of 11.8% (6/51), a reoperation rate of 9.8% (5/51), and a subsequent TKA rate of 5.9% (3/51), and 100% (3/3) of subsequent TKAs had an existing medial cartilage defect at the time of repair (Table 2), whereas only 33.3% (1/3) had an existing lateral cartilage defect. Repairs with existing chondral pathology analyzed by sex (female and male, respectively) had a clinical retear rate of 5.3% (2/38) and 30.8% (4/13), a reoperation rate of 5.3% (2/38) and 23.1% (3/13), and a subsequent TKA rate of 2.6% (1/38) and 15.4% (2/13). Repairs without any chondral pathology had a clinical retear rate of 0% (0/12), a reoperation rate of 0% (0/12), and a subsequent TKA rate of 0% (0/12; Table 2). There was no statistically significant difference in clinical retear rate (11.8% vs 0%, *P* = 1.000), reoperation rate (9.8% vs 0%, *P* = 1.000), or subsequent TKA rate (5.9% vs 0%, *P* = .476) with or without existing chondral pathology at the time of repair, respectively. 71.9% (41/57) of repairs that did not fail had at least one associated procedure (Table 3). One hundred percent (6/6) of repairs that failed had at least one associated procedure (Table 3).

**TABLE 4:** Count and Percentage of Isolated Meniscus Root Repairs With Chondral Pathology (i.e., Erosion, Full‐Thickness Lesions, Damage, Superficial Fraying, Superficial Loss, or Chondromalacia) Present at the Time of Surgery (Grouped by Knee Compartment, M, L, PF, %) as Reported by the Operating Surgeon With Direct Visualization During Arthroscopy (n = 51)

Chondral Pathology	Total	M Only	L Only	PF Only	M&L Only	M&PF Only	L&PF Only	M&L&PF
Count	51	16	0	1	4	25	1	4
Knee Compartment		**M**			**L**		**PF**	
%		96.1% (49/51)			17.6% (9/51)		60.8% (31/51)	

%, percentage of repairs with chondral defects; M, medial; L, lateral; PF, patellofemoral.

## DISCUSSION

In this study, we found that there were no statistically significant differences between isolated meniscal root repair outcomes with and without chondral pathology; however, there was a trend of increased failure outcomes associated with chondral pathology present at the time of the index surgery. Of the 63 isolated meniscal root repairs in this study, 51/63 (81%) had existing chondral pathology at the time of surgery, and 49/51 (96.1%) of those had chondral pathology involving the medial compartment of the knee. Despite our results not achieving statistical significance regarding a difference in meniscal root retear, reoperation, or subsequent TKA conversion rates in the chondral pathology versus no chondral pathology cohorts, we did identify that all repair failures took place within the chondral pathology cohort. The lack of statistical significance can likely be attributed to the small sample size. It is possible that with an extended database, this could yield a statistically significant finding. But considering there is a paucity of data looking specifically at the effects of chondral pathology of the knee in the setting of isolated meniscal root repairs, this associated trend we discovered can help surgeons guide clinical decision‐making to decrease retear rate and the need for subsequent operations by looking into this topic further in the future.

Chondral pathology has previously been reported in over 60% of patients undergoing knee arthroscopy.^8^, ^9^ In addition, surgery for meniscal pathology has previously been reported as the most common orthopaedic surgery performed in the United States.^1^, ^10^ Chambers et al.^11^ found that meniscal root tears increased the risk of developing osteoarthritis when compared to nonroot tears. Zhan et al.^12^ previously reported that medial meniscus tears (root and radial) were associated with an increased risk of medial meniscal extrusion, defined as protrusion of the medial meniscus beyond the tibial edge by more than 3 mm. Meniscal extrusion has also been found to increase the risk of cartilage damage, and osteoarthritis. In this study, 81% of patients who underwent isolated meniscal root repairs had existing chondral pathology.

Although the association between meniscus and chondral pathology has been well described, there is a lack of data regarding the effect that chondral pathology has on outcomes and healing after meniscus repair. Jiang et al.^13^ completed a systematic review to determine what clinical factors were associated with successful meniscal root repairs and identified high‐grade (Outerbridge grade ≥3) chondral lesions and varus alignment >5° to be associated with poor clinical outcomes for medial posterior root repairs. Helito et al.^14^ also identified female gender, high BMI, concomitant axial alignment correction, pronounced chondral injury, the use of 3 repair stitches, and poor patient compliance to be risk factors for failure of medial meniscus posterior root repairs.

Therefore, we hypothesized that existing chondral pathology would be associated with increased isolated meniscus root repair retear rates. However, although our analysis showed no significant difference, a trend was observed as all repair failures occurred in patients with existing chondral pathology at the time of repair, but we acknowledge that this could also be due to factors not specifically focused on in our study (i.e., the size and grade of cartilage injury, or other differences between meniscal repair patients). This information can help guide clinical decision‐making for providers and guide future research for optimizing surgical decision‐making and treatment techniques. As tissue quality plays a role in the success of surgical repair, it could have been informative to analyze whether the failures in our study were traumatic injuries or degenerative injuries.

It is notable that of 574 meniscus repair procedures at this institution over 10 years, only 63 isolated meniscal root repairs were performed, and all of the isolated meniscal root repair cases involved the posterior root of the medial meniscus. The majority of cases that were excluded were due to the fact that they were performed in conjunction with other procedures such as meniscectomy, cartilage restoration, osteotomy, or other concurrent ligament repair or reconstruction. This is relevant especially because lateral meniscus root tears are 10.3 times (95% CI 2.6‐42.5) more likely to have a concomitant anterior cruciate ligament tear than patients with medial meniscus root tears (*P* = .012)^7^ and would help explain why our results are far more weighted towards medial meniscus tears (63/63 medial, 100%). Matheny et al.^7^ also found that patients with medial meniscus root tears were 5.8 times (95 % CI 1.6‐20.5) more likely to have chondral defects than patients who had lateral meniscus root tears (*P* = .044) which also aligns with the 81.0% (51/63) of repairs in our study that had an existing cartilage defect at the time of repair.

### Limitations

This study is not without limitations. The retrospective nature of this study creates a risk for information and selection biases. The small sample size creates the potential for a type II error. Furthermore, the lack of analysis of the effect of cartilage injury size, depth, and grade is also a limitation of this study, as it prevents it from being directly compared with current literature using these measures. Another limitation is the lack of an established minimum follow‐up time, as we included subjects with less than 1 year of follow‐up. By not having a predetermined minimum time, there was bias toward repairs completed earlier in the 2012 to 2022 timeframe to have a longer period to retear or require a subsequent reoperation. In addition, we did not record or exclude procedures based on the etiology of the tears (degenerative vs trauma), which may also affect retear rates.

## CONCLUSIONS

In this study, we did not find a statistically significant difference in meniscal root retear, reoperation, or subsequent TKA rates with or without existing chondral pathology.

## DISCLOSURES

The authors (K.R.D., B.R.W., R.W.W.) declare the following financial interests/personal relationships which may be considered as potential competing interests: K.R.D. reports funding grants from Orthopaedic Research and Education Foundation and board membership with Video Journal of Sports Medicine. B.R.W. reports funding grants from Arthritis Foundation; consulting or advisory fees and equity or stocks from CONMED Corporation; and board membership with The American Board of Orthopaedic Surgery, American Orthopaedic Society for Sports Medicine, and Mid‐America Orthopaedic Association. R.W.W. reports consulting or advisory fees from Smith & Nephew, CONMED Corporation, and Responsive Arthroscopy, and board membership with American Orthopaedic Society for Sports Medicine, Arthroscopy Association of North America, and International Society for Hip Arthroscopy. The other authors (M.D.B., R.J.V., A.C.B., B.D.P., S.M.L.) declare that they have no known competing financial interests or personal relationships that could have appeared to influence the work reported in this article.

## References

[1] Li J , Zhu W , Gao X , Li X . Comparison of arthroscopic partial meniscectomy to physical therapy following degenerative meniscus tears: A systematic review and meta‐analysis. Biomed Res Int. 2020;2020:1709415. 32190650 10.1155/2020/1709415 PMC7073498

[2] Calanna F , Duthon V , Menetrey J . Rehabilitation and return to sports after isolated meniscal repairs: A new evidence‐based protocol. J Exp Orthop. 2022;9:80. 35976500 10.1186/s40634-022-00521-8 PMC9385921

[3] Jarraya M , Roemer FW , Englund M , et al. Meniscus morphology: Does tear type matter? A narrative review with focus on relevance for osteoarthritis research. Semin Arthritis Rheum. 2017;46:552‐561. 28057326 10.1016/j.semarthrit.2016.11.005

[4] Krych AJ , Hevesi M , Leland DP , Stuart MJ . Meniscal root injuries. J Am Acad Orthop Surg. 2020;28:491‐499. 31693530 10.5435/JAAOS-D-19-00102

[5] Schweizer C , Hanreich C , Tscholl PM , et al. Nineteen percent of meniscus repairs are being revised and failures frequently occur after the second postoperative year: A systematic review and meta‐analysis with a minimum follow‐up of 5 years. Knee Surg Sports Traumatol Arthrosc. 2022;30:2267‐2276. 34671817 10.1007/s00167-021-06770-x PMC9206598

[6] Allaire R , Muriuki M , Gilbertson L , Harner CD . Biomechanical consequences of a tear of the posterior root of the medial meniscus: Similar to total meniscectomy. J Bone Joint Surg Am. 2008;90:1922‐1931. 18762653 10.2106/JBJS.G.00748

[7] Matheny LM , Ockuly AC , Steadman JR , LaPrade RF . Posterior meniscus root tears: Associated pathologies to assist as diagnostic tools. Knee Surg Sports Traumatol Arthrosc. 2015;23:3127‐3131. 24866130 10.1007/s00167-014-3073-7

[8] Hjelle K , Solheim E , Strand T , Muri R , Brittberg M . Articular cartilage defects in 1000 knee arthroscopies. Arthroscopy. 2002;18:730‐734. 12209430 10.1053/jars.2002.32839

[9] Curl WW , Krome J , Gordon ES , Rushing J , Smith BP , Poehling GG . Cartilage injuries: A review of 31,516 knee arthroscopies. Arthroscopy. 1997;13:456‐460. 9276052 10.1016/s0749-8063(97)90124-9

[10] Cullen KA , Hall MJ , Golosinskiy A . Ambulatory surgery in the United States, 2006. Natl Health Stat Report. 2009;11:1‐25. 19294964

[11] Chambers CC , Lynch JA , Feeley BT , Nevitt MC . Association of medial meniscus root tears and nonroot tears with worsening of radiographic knee osteoarthritis. Orthop J Sports Med. 2023;11:23259671231195894. 37711506 10.1177/23259671231195894 PMC10498710

[12] Zhan H , Liu Z , Wang Y , et al. Radiographic OA, bone marrow lesions, higher body mass index and medial meniscal root tears are significantly associated with medial meniscus extrusion with OA or medial meniscal tears: A systematic review and meta‐analysis. Knee Surg Sports Traumatol Arthrosc. 2023;31:3420‐3433. 37099153 10.1007/s00167-023-07418-8

[13] Jiang EX , Abouljoud MM , Everhart JS , et al. Clinical factors associated with successful meniscal root repairs: A systematic review. Knee. 2019;26:285‐291. 30772183 10.1016/j.knee.2019.01.005

[14] Helito C , Sobrado M , Guimaraes T , Silva da A , Pecora J , Gobbi R . Defining risk factors for medial meniscus posterior root repair surgical failure. Orthop J Sports Med. 2024;12(11_suppl4):2325967124S00516.

